# Profile of Folate in Breast Milk from Chinese Women over 1–400 Days Postpartum

**DOI:** 10.3390/nu14142962

**Published:** 2022-07-20

**Authors:** Yanyan Su, Yingyi Mao, Fang Tian, Xiaokun Cai, Ruidi Chen, Na Li, Changli Qian, Xiang Li, Yanrong Zhao, Yu Wang

**Affiliations:** 1 School of Public Health, Lanzhou University, Lanzhou 730000, China; suyy19@lzu.edu.cn (Y.S.); chenrd19@lzu.edu.cn (R.C.); lin14@lzu.edu.cn (N.L.); qianchl14@lzu.edu.cn (C.Q.); 2 Abbott Nutrition Research & Development Center, Abbott Ltd., Shanghai 200233, China; yingyi.mao@abbott.com (Y.M.); fang.tian@abbott.com (F.T.); cxkun1990@outlook.com (X.C.); xiang.li@abbott.com (X.L.)

**Keywords:** MUAI, Chinese breast milk, folate, 5-methyl-tetrahydrofolate

## Abstract

Folate is an essential nutrient for growth in early life. This study aimed to determine the levels and compositions of folate in Chinese breast milk samples. This study was part of the Maternal Nutrition and Infant Investigation (MUAI) study. A total of 205 healthy mothers were randomly recruited in Chengdu over 1–400 days postpartum. Five different species of folate, including tetrahydrofolate (THF), 5-methyl-THF, 5,10-methenyl-THF,5-formyl-THF and unmetabolized folic acid (UMFA), were measured for liquid chromatography–tandem mass spectrometry (LC-MS). The median levels of total folate ranged from 12.86 to 56.77 ng/mL in the breast milk of mothers at 1–400 days postpartum, gradually increasing throughout the lactating periods. The median levels of 5-methyl-THF, minor reduced folate (the sum of THF, 5,10-methenyl-THF and 5-formyl-THF) and UMFA were in the ranges of 8.52–40.65 ng/mL, 3.48–16.15 ng/mL and 0.00–1.24 ng/mL during 1–400 days postpartum, respectively. 5-Methyl-THF accounted for more than 65% of the total folate in all breast milk samples. The levels of UMFA in mature breast milk samples were higher in supplement users than nonusers, but not for colostrum and transitional milk samples (*p* < 0.05). In conclusion, the level of total folate in the breast milk changed along with the prolonged lactating periods, but 5-methyl-THF remains the dominant species of folate in the breast milk of Chinese populations across all entire lactating periods.

## 1. Introduction

Breast milk is a preferable source of nutrients to ensure infants’ optimal growth, health and development during early-life [1]. The World Health Organization recommends that breast milk is the best source of early nutrition for infants from birth to at least 6 months of age, and that breast milk should continue to be an important part of the diet of infants until 2 years old [2]. Folate is an essential nutrient for humans that functions as a family of metabolic cofactors that participate in the synthesis of purine and pyrimidine, cellular methylation reactions, 1-carbon transfer reactions, amino acid metabolism and myelination and neurotransmitter synthesis [3,4]. During the embryonic and fetal periods, the lack of folate has been linked to an increased risk of numerous negative reproductive outcomes such as neural tube defects (NTDs), small for gestational age and preterm delivery [5,6,7]. Studies have also shown that folate may promote neural development in the brain during infancy and early childhood, which are sensitive and rapid periods of brain growth [8,9,10].

Folate, a member of the B-vitamin group, consists of a common parent structure, but it differs with an oxidized or reduced state of the pteridine ring, the one-carbon substituents (methyl, formyl, methylene, methenyl or formimino) at position N5 and N10 and the length of their glutamate tails [11]. Folate is naturally one of the reduced polyglutamate species, including 5-methyl-tetrahydrofolate (5-methyl-THF), tetrahydrofolate (THF), 5,10-methenyl-tetrahydrofolate (5,10-methenyl-THF) and 5-formyl-tetrahydrofolate(5-formyl-THF) [12,13]. Folic acid, conjugating one glutamic acid residue, is a synthetic and oxidized species of the folate family. Since folic acid is more stable than other species, it is commonly used as a nutrient fortifier in infant formulas, dietary supplements and fortified foods [4,14]. However, folic acid is inactive in the human body and need to be transferred to tetrahydrofolate by the dihydrofolate reductase (DHFR) in the liver [15]. The activity of human hepatic DHFR is thought to be low and quite variable between individuals. In vitro, this enzyme tends to get saturated by quantities of folic acid above 0.4 mg [16]. The concentrations > 1 mol/L of unmetabolized folic acid (UMFA) in plasma were largely explained by the intake of total folic acid from diets and supplements [17]. The evidence from animal studies and population epidemiological studies suggests that a high concentration of UMFA may have adverse consequences [18]. Animal studies have shown that a high intake of synthetic folic acid may lead to epigenetic changes in offspring, increasing the risk of diabetes and altering food intake behavior [19]. The Boston Birth Cohort Study [20] showed that the level of UMFA in the umbilical cord blood was positively associated with an increased risk of autism spectrum disorder in black children (OR = 9.85; 95% CI: 2.53, 38.31).

Breast milk contains different species of folate, and the level of total folate changes along with the lactating periods. The folates are naturally occurring in reduced forms and in synthetic form as folic acid. It can be observed that breast milk folate increases as lactating proceeds. The concentration of total folate ranges from 15.1 to 17.0 ng/mL in colostrum and increases to 26.2 to 42.0 ng/mL in mature milk [21,22]. Additionally, folic acid supplement may impact the concentrations and distributions of different folate species in breast milk. A recent study [12] showed that the total folate in breast milk was significantly higher in women who consumed folic acid supplements. However, the higher level of total folate was due to the increase in UMFA concentrations but not to reduced folates. 5-Methyl-THF was significantly lower in milk from women who consumed folic acid supplements (>400 µg/d). Given the higher affinity of the folate-binding protein (FBP) for folic acid and the less mature gastrointestinal system in infants, folic acid may not be as readily released from FBP in the infant gastrointestinal tract [23]. Changes in the distribution of folate vitamers could possibly impact the bioavailability of breast milk folate and further impact folate absorption in infants.

Currently, although some studies have reported the concentrations of total folate in breast milk, the content and distribution of folate species in breast milk in Chinese lactating mothers during the first year postpartum have not been reported. The aim of this study was to profile the folate in breast milk at 1–400 days postpartum. To our knowledge, this was the first study exploring the concentrations of various milk folate species over 1–400 days postpartum in a healthy Chinese population.

## 2. Material and Methods

### 2.1. Participants and Human Milk Sample Collection

This cross-sectional study was part of the Maternal Nutrition and Infant Investigation (MUAI). From March 2018 to June 2019, 205 healthy women aged 20–35 years were recruited at Chengdu Women’s and Children’s Central Hospital, Chengdu, China. Breast milk samples were collected at five different time points: 1–5 days, 10–15 days, 40–45 days, 200–240 days and 300–400 days postpartum, covering 3 lactation stages, colostrum (1–5 days), transitional milk (10–15 days) and mature milk (40–400 days). This study also collected maternal and infant information and supplement-use questionnaires at each sampling time, which were conducted by doctors and trained students. The inclusion criteria were: the mother had lived in the region for more than two years, had a singleton and had a full-term delivery. Thirty mothers who donated colostrum (1–5 days), transitional milk (10–15 days) and mature milk (40–45 days) were included who planned to exclusively breastfeed their infant for more than two months. All mothers were medically certified as healthy and well-nourished, as assessed by nutritionists through a nutritional assessment, including a dietary assessment, anthropometric measurement, physical examination and biochemical tests. Their infants were appropriate for gestational age newborn infants and had an Apgar score greater than 8. Women were excluded from participation if they had a chronic disease, acute or chronic infectious disease or any other diseases affecting nutrient metabolism. The MUAI study was registered at the Chinese Clinical Trial Center (ChiCTR1800015387). All procedures were approved by the Ethics Committee of Chengdu Women’s and Children’s Central Hospital (IEC-C-005-V.03). All mothers who participated in the project gave informed written consent. All procedures performed in the study that involved human participants followed the ethical standards of the institutional or national research committee or both and in line with the 1964 Helsinki Declaration and its later amendments or comparable ethical standards.

All mothers were required to follow a uniform sampling procedure. A single full breast (08:00–11:00 a.m.) was emptied by hand or with an electric pump. The collecting tube was turned upside down 6 times to mix the breast milk well, and 30–50 mL of breast milk was poured into a sterile, light-avoiding centrifugal tube (AS ONE TB1500 and AS ONE TB5000), except for colostrum milk (8–10 mL) and transitional milk (10–30 mL). The remaining milk was returned to feed the baby. The milk sample were transported to the laboratory via a cold chain within five hours, then each milk sample was distributed into 1.2 mL cryogenic vials (Corning 430658) under yellow light, labelled with subject information and immediately frozen at −80 °C. A total of 205 human milk samples were collected from 205 mothers at five time periods.

### 2.2. Quantification of Folate

Breast-milk folates were measured with the use of liquid chromatography–tandem mass spectrometry (LC-MS). The method analyzed the four most prevalent species of naturally occurring folate vitamers as well as synthetic folic acid: 5-methyl-THF, THF, 5,10-methenyl-THF, 5-formyl-THF and UMFA, respectively [12,24,25]. In brief, breast milk (500 µL) was diluted in 2 mL SPE conditioning buffer that contained 1% ammonium acetate, 2% ascorbic acid and 0.1% dithiothreitol. Then, 20 µL internal standard solution (0.5 µg/mL of 5-methyl-THF-(glutamic acid-13C5), 5 µg/mL of folic acid-(glutamic acid-13C5)) was added. The samples were incubated (2 h, 37 °C) with 100 µL of rat serum (to deconjugate the polyglutamate forms of folates to monoglutamates), defatted with 1 mL chloroform and centrifuged (5 min, 3000× *g*). Then, sample cleanup was performed on Oasis MAX (60 mg, 3 mL) solid-phase extraction (SPE) cartridges preconditioned with 2 mL acetonitrile followed by 2 mL SPE conditioning buffer. The supernatant was loaded on the cartridge and passed through the sorbent bed, and impurities were removed by washing the columns with 2 mL SPE conditioning buffer. The folate species were eluted into a 15 mL centrifuge tube with 2 mL SPE elution buffer that contained 60% (v) acetonitrile and 40% (v) SPE conditioning buffer. Evaporation occurred under N_2_ gas at 55 °C to a final volume of ca. 0.5 mL. All samples were processed under yellow light.

The purified extract was analyzed in positive-ion mode using electrospray ionization via LC (Agilent technologies 1290 infinity II)-MS (AB Sciex API 5500). The injection column volume was 10 µL. Chromatographic separation was achieved using an Acquity UPLC^®^ BEH C18 1.7 µm (2.1 × 100 mm Column, waters) analytic column with a gradient elution, using ammonium acetate in water (20 mmol/L, eluent A) and methanol (eluent B) as the mobile phases at 0.35 mL/min and a total run time of 10 min. The gradient used for the analysis of folate was as follows: 0.00 min, 0% A, 100% B; 0.50 min, 0% A, 100% B; 2.00 min, 8% A, 92% B; 2.50 min, 8% A, 92% B; 5.00 min, 100% A, 0% B; 6.00 min, 100% A, 0% B; 7.00 min, 0% A, 100% B; 10.00 min, 0% A, 100% B. Quantitation was achieved with the use of stable-isotope-labelled internal standards and an external standard calibration curve. The samples were analyzed in duplicate. The sum of these folates was reported as the total folate. Due to the possibility of mutual transformation among 5,10-methenyl-THF, THF and 5-formyl-THF, the contents of these three species of folate were combined in this study and called the minor reduced folate. The reduced folates represented the sum of the minor reduced folate and 5-methyl-THF. The limit of detection (LOD) values of the method were 0.06 ng/mL for 5-methyl-THF, 0.04 ng/mL for THF, 0.01 ng/mL for 5,10-methenyl-ThF, 0.05 ng/mL for 5-formyl-THF and 0.16 ng/mL for UMFA, respectively.

### 2.3. Statistical Analysis

Statistical analyses were performed with the use of SPSS software 23.0 (SPSS Inc. Chicago, IL, USA). The demographic characteristics of lactating women were described as the count (percentage) for categorical variables and the mean ± standard deviation for continuous variables. One-way ANOVA (continuous variables) and Chi-squared tests (categorical variables) were used to compare participants’ characteristics according to periods of lactation. The median values with the interquartile range were calculated for concentrations of breast milk folate according to the research periods of 0–5 days postpartum, 10–15 days postpartum, 40–45 days postpartum, 200–240 days postpartum and 300–400 days postpartum. Due to the nonnormal distribution in human milk folate, the Kruskal–Wallis test was used to analyze the content of breast milk in five lactating periods. Bonferroni correction was used for multiple comparisons if there were differences between groups. The two-independent nonparametric test (Kolmogorov–Smirnov Z) was used to compare the concentrations of breast milk folate between supplement nonusers and users. Here, *p* < 0.05 was considered statistically significant.

## 3. Results

### 3.1. Basic Characteristics

The basic information for the research participants is shown in Table 1. A total of 205 breast milk samples were collected. The average age of the subjects was 29.1 ± 3.1 years, and the BMI values before pregnancy and in the prenatal period were 20.6 ± 2.5 kg/m^2^ and 26.2 ± 2.8 kg/m^2^, respectively. The average weight gain during pregnancy was 14.4 ± 4.9 kg. There were no differences in age, prepregnancy BMI, predelivery BMI, gestational weight or offspring gender among different lactating periods (*p* > 0.05). The mean gestational age was 39.5 ± 1.7 weeks. The Apgar scores for all newborns were greater than 8. There was no difference in the daily consumption between summer and winter groups over the last month of common food (Supplementary Materials Table S1).

### 3.2. Milk Folate Levels over Lactational Periods

**Figure 1:**
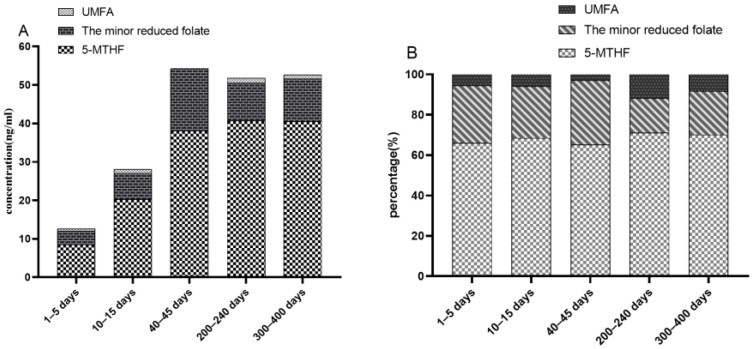
The concentrations of breast milk folate from 1 to 400 days postpartum (**A**). The distribution of folate species over 1–400 days postpartum (**B**). The minor reduced folate represents the sum of THF, 5,10-methenyl-THF and 5-formyl-THF; 5-MTHF, 5-methyltetrahydrofolate; UMFA unmetabolized folic acid.

The changes in folate levels over 1–400 days postpartum are shown in Table 2 and Figure 1A. The median concentrations of total folate, reduced folate and 5-methyl-THF were observed to be lower in colostrum and transitional milk than in mature milk at 40–45 days, 200–240 days and 300–400 days (*p* < 0.05). No difference was observed in the three periods of mature milk. The median concentration of the minor reduced folate in the colostrum was observed to be lower than in transitional milk and mature milk at 40–45 days (*p* < 0.05). The concentrations of UMFA in the mature milk samples at 40–45 days were significantly lower than breast milk samples during other lactational periods (*p* < 0.05). Concentration of breast milk folate over 1–400 days postpartum of supplement nonusers and users were showed in supplementary materials Tables S2 and S3, respectively.

The proportions of different folate species at different lactating periods are shown in Figure 1B. Throughout 1–400 days postpartum, 5-methyl-THF was the predominant species of folate in breast milk samples (65.1~70.9%). The minor reduced folate was in second place (17.0~31.9%), while UMFA accounted for the smallest proportion (3.0~12.1%).

### 3.3. Supplement Users Compared with Nonusers

**Figure 2:**
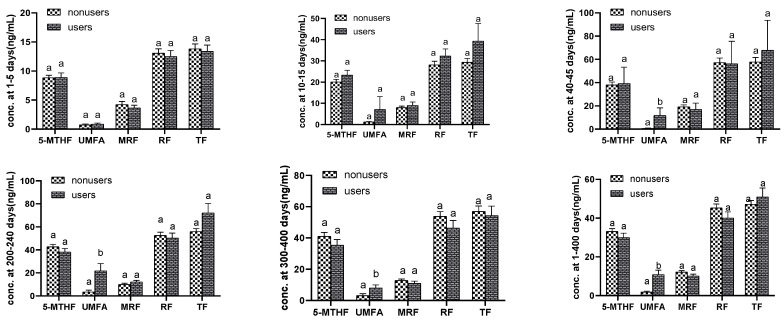
Mean ± SE 5-MTHF, UMFA, minor reduced folate (MRF), reduced folate (RF) and total folate (TF) contents in the breast milk of Chinese women, in women who were folic acid-supplement nonusers and users at 1–5 day, 10–15 day, 40–45 days, 200–240 day, 300–400 days and 1–400 days postpartum, respectively. Note: *p* values (2-sided) were derived by using the two-independent nonparametric test (Kolmogorov–Smirnov Z). Values that do not share a lowercase letter are significantly different; *p* < 0.05 was considered significant.

Supplement users were defined as women who consumed synthetic folic acid as either a multivitamin or a single folic acid supplement during the lactating period. Of the 205 participants, 29.3% of subjects were supplement users and 70.7% of subjects were supplement nonusers. Here, 36.7%, 26.7%, 12%, 33.3% and 39.1% of mothers from lactation periods of 1–5 days, 10–15 days, 40–45 day, 200–240 days and 300–400 days were supplement users, respectively. A comparison of the breast milk folate concentrations between users and nonusers is shown in Figure 2. Differences in the concentrations of UMFA were observed between supplement nonusers and users at 40–45 days, 200–240 days and 300–400 days postpartum (*p* < 0.05). The total folate, reduced folates, the minor reduced folate and 5-methyl-THF concentrations in breast milk were not significantly different between supplement nonusers and users throughout 1–400 days postpartum (*p* > 0.05).

## 4. Discussion

To our knowledge, this study was the first to report the concentrations of different folate species over 1–400 days postpartum in a Chinese population. Our study measured five major folate species in Chinese breast milk samples and investigated the changes in total folate as well as in each species of folate during the lactating periods.

The concentrations of total folate in breast milk samples ranged from 12.86 to 56.77 ng/mL at 1–400 days postpartum, which were different from the data from some other studies. This study reported higher folate levels than an observation study involving 443 Chinese lactating mothers, which showed that the folate concentrations in breast milk were 7.30–24.40 ng/mL over 5–240 days postpartum [26]. Additionally, the total folate concentrations ranged from 88.80 to 161.33 ng/mL in the first 6 months postpartum in Korean women [27]. The total folate in breast milk at 40–45 days postpartum (54.65 ng/mL) from our study was similar to the level (52.60 ng/mL) given by Page as representing breast milk at approximately 40 days postpartum [12]. The differences in total folate concentrations of breast milk among the different studies may be related to the quantitative methods and the procedures of milk collection and storage. There were a variety of methods for the measuring of breast milk folate concentrations, such as microbiological assays, LC-MS and competitive radio- and chemiluminescence assays [28]. Currently, most published data have been obtained with microbiological assays, which can report the total folate levels but without distinguishing individual species [29]. Additionally, large differences in accuracy among different folate assays have been observed [29]. Recently, LC-MS has also been recommended to determine the concentrations of individual folate species in breast milk [12]. Therefore, our study was optimized and validated as an LC-MS-based method to measure the folate in Chinese breast milk, which showed a high spiked recovery rate and RSD values for parallel samples. Moreover, increases in milk folate concentrations with the time of day and from foremilk to hindmilk were observed in several studies [30]. In addition, the conditions of milk storage can also impact the folate concentration. Bank et al. showed that folate concentrations in breast milk decreased with the extension of frozen storage (−20 °C) [31]. In this study, the standard milk collection method was shown to participants by trained interviewers and consistent timing of collection was required for each sample.

Our results also showed that the folate levels in breast milk varied significantly among each lactating period. The concentrations of breast milk increased from colostrum to transitional milk and remained gradually stable in mature milk. Previous studies also [21,27,32] showed that total folate in breast milk changed with the progression of lactation, which increased from colostrum milk to mature milk. There were two possible reasons to the change the total folate levels along the lactation course: (1) Folate can accumulate in the fetus since the placenta actively transfers folate from the mother to the fetus [33]. Several studies have reported that cord blood folate was higher than that in maternal blood [34,35], suggesting that newborns had the stores of folate in their body. (2) Infants demands on folate increase along with their growth and development. However, some studies observed a decline in the breast milk folate concentration during later lactation [27,36]. Longitudinal studies on the folate concentrations of breast milk in the Chinese population are needed in the future for a better understanding of the changes in breast milk over the lactation period.

5-Methyl-THF was the predominant folate form in breast milk at different lactating periods. In this study, 5-methyl-THF (8.52~40.65 ng/mL) accounted for the largest proportion of total folate content (65~71%) in all breast milk samples. An observation study [12] involving 561 Canadian lactating mothers at approximately 40 days postpartum, whose mean total daily intake of folic acid from supplements was 979 µg/day, showed that the 5-methyl-THF concentration in breast milk was 49.7 nmol/L (22.8 ng/mL), which was lower than in our study (38.06 ng/mL, 40~45 days postpartum). The author also observed that 5-methyl-THF represented 53–55% of the total milk folate in 203 lactating mothers who had a daily folic acid supplement intake of <400 µg/day. 5-Methy-THF is the bioactive form of folate, which can be directly transported into the circulation after absorption [14], and primarily participates in the regeneration of methionine from homocysteine and further enters the methylation cycle [4]. A recent clinical study reported that healthy term infants (*n* = 240) fed with formula containing 5-methyl-THF or folic acid showed no differences in the occurrence of adverse events, weight gain or gain in head circumference. However, infants receiving formula containing 5-methyl-THF had lower UMFA levels in plasma (0.73 vs. 1.15 nmol/L, *p* < 0.0001) than infants fed with folic-acid-fortified formula [37]. As supported by its safety, high proportion in breast milk and clinical studies, 5-methyl-THF has already been approved as a source of folate in infant formulas by the EFSA [38]. The reduced folate accounted for 87.9~97.0% of the total folate in breast milk at all lactating periods, in which 17.0~31.9% was the minor reduced folate. Rachael Page et al. [12] reported that the minor reduced folate in breast milk of mothers at approximately 40 days postpartum represented 18~22% of the total folate, which was lower than our result (31.9%, 40~45 days postpartum). The intracellular folate metabolism is complex. Normally, after absorption, the minor reduced folate (THF, 5-formyl-THF and 5,10-methenyl-THF) can be transformed to 5-methyl-THF during passage through the intestinal cells and then transferred to the circulation [14,39]. The minor reduced folate also is biologically active [14]. Formyl-THF donates formyl groups for purine synthesis, which is an essential step in DNA synthesis. 5,10-Methylene-THF is the coenzyme for thymidylate synthesis, which is involved in the addition of a formaldehyde group to deoxyuridine monophosphate (dUMP), leading to the formation of deoxythymidine monophosphate (dTMP) [4,40].

Here, he concentrations of UMFA were significantly lower than the reduced folate in all breast milk samples. Our result showed that the median concentrations of UMFA ranged from 0.00 to 1.24 ng/mL, representing 3.0%~12.1% of the total folate in breast milk samples from 1 to 400 days postpartum. Houghton’s study showed that the UMFA concentration in breast milk samples at 16 weeks postpartum was 6.4 ± 4.3 µg/L and represented 8% of the total folate [41], which was similar to our study. Moreover, Page reported that the median UMFA concentration in breast milk samples at approximately 40 days postpartum was 47.0 ± 1.6 nmol/L (20.7 ng/mL), and represented 45% of the total folate in folic acid supplement users and 23% in nonusers [12]. Another observational study also showed that UMFA (24.1 ng/mL) accounted for 40% of the total folate in breast milk samples from women who consumed a 750 ug folic acid supplement/d for 10 weeks [42]. These studies suggested that the folic acid intake may lead to the difference in UMFA concentration and the distribution of folate in breast milk. It is worth noting that 70.7% of subjects in this study were nonusers. Our report also observed that supplement users during the lactating period had higher UMFA concentrations in breast milk than nonusers at 40–45 days, 200–240 days and 300–400 days postpartum. The MTHFR 677TT genotype was reported to be associated with the UMFA concentration in breast milk (R^2^ = 0.01; unadjusted *p* = 0.004) [43]. The enzyme methylenetetrahydrofolate reductase (MTHFR) plays a key role in regulating the folate cycle, in which 5,10-methenyl-THF was irreversibly reduced to 5-methyl-THF. The common C677T variant (rs1801133) in the gene that encodes MTHFR is a C-to-T transition at position 677, resulting in a mildly dysfunctional thermolabile MTHFR enzyme and leading to a 30% decrease in enzyme activity in heterozygotes and a 60% decrease in homozygotes [44]. The study showed that the decrease in MTHFR enzyme activity was associated with the decrease in folate concentrations and the increase in homocysteine [45]. TT homozygotes require higher folate intakes than do individuals with the CT or CC genotype to achieve similar homocysteine concentrations [46]. For people carrying MTHFR 677TT, excessive folic acid intake can lead to the so called UMFA syndrome, which may have highly deleterious consequences. The folate level is reported in Chinese populations using either microbial methods and a test kit for the content or the change trends and distribution patterns of sub-species of folate associated with the MTHFR 677TT genotype. There is a need to analyze the breast milk levels in the Chinese population based on 5-methyl-THF and UMFA. Wilcken et al. reported the varied prevalence of the MTHFR 677TT genotype across ethnic groups and regions, and the content and distribution pattern of these folate species is worthy of further comparison [47]. Therefore, this study could provide Chinese population data to compare with Canadian studies to demonstrate that 5-methyl-THF is predominate despite the genetic and dietary supplement intake differences.

The results of this study also showed that the folic acid supplementation impacted the concentration of UMFA in breast milk at 40–400 days postpartum. The impact of maternal folic acid supplement intake on breast milk folate levels remains controversial. Several studies reported that consuming a folic acid supplement at 400–1000 µg/day could maintain the maternal folate status but does not result in higher total milk folate [39,42]. Previous studies conducted in Canada have shown that 400 µg/day of folic acid intake did not impact the total folate in breast milk, while more than 400 µg/day folic acid supplement decreased the level of 5-methyl-THF but increased the total folate in breast milk [12,41]. In China, a study involved 954 participants from 13 provinces and municipalities and reported that the folate intake (including food folate and supplemental folic acid) of lactating mothers was 212.27 µg/day, which was much lower than 400 µg/day [48]. Presumably, Chinese mothers have a very low intake of folic acid and less UMFA in their breast milk. A small trial showed that the total folate concentration in breast milk increased significantly in folic acid users who were folate-deficient and in low socioeconomic lactating women [49]. In addition, the dietary intakes showed no differences between user and nonuser groups, indicating that genetic variation and supplement intake should be considered as major impacts in this study, which are also worthy of future investigation in terms of the dietary impacts. Given that 5-methyl-THF was the predominant folate species in breast milk, more studies on 5-methyl-THF supplementation in the maternal population should be carried out in the future. In addition, free folic acid needs to be differentiated from the folate levels in breast milk, as free folic acid is mainly introduced via supplementary intake or the oxidation of reduced folates, meaning it can be traceable or below the LOQ in the breast milk of supplement-nonuser donors [50]. For this reason, this study used rat serum as the source of deconjugase to convert the polyglutamate forms into the monoglutamate form to ensure the complete extraction of all folate species in breast milk.

The limitations of the present study should be noted. Firstly, the present study was an observational design milk sample collection at one time point for one participant and may limit the ability for speculation regarding long-term changes of nutrient constituents along lactating periods. Secondly, the data on maternal folate intakes (including dietary and folic acid supplements) were not reported.

## 5. Conclusions

This study measured the levels of five major folate species, namely 5-methyl-THF, THF, 5,10-methenyl-THF, 5-formyl-THF and UMFA, in Chinese breast milk samples during 1–400 days postpartum. Over the course of lactation, the concentrations in total folate were much higher in mature milk than colostrum and transitional milk. 5-Methyl-THF was the dominant species of folate in breast milk throughout the entire lactation period, suggesting that 5-methyl-THF is the optimal species of folate for infants. The intake of folic acid increased the concentrations of UMFA in mature milk, which requires more studies to understand the impacts of higher UMFA levels on infant growth. Our study firstly reported the profiles of folate in Chinese breast milk in different lactating periods, and 5-methyl-THF was continuously reported as the predominant folate type. In addition, as a periodical review of folate in Chinese breast milk, this study could provide a scientific reference for optimizing the folate content and form for Chinese DRI updates and scientific evidence on the fortification level and type of folate in infant formula.

## Figures and Tables

**Table 1:** Characteristics of participant mothers and corresponding infants at each sampling time.

	Days Postpartum (*n*)					*p* Value
	1–5 Days (*n* = 30)	10–15 Days (*n* = 30)	40–45 Days (*n* = 48)	200–240 Days (*n* = 51)	300–400 Days (*n* = 46)	
Age (years)	28.3 ± 3.3	29.0 ± 2.2	29.6 ± 2.8	29.0 ± 3.8	29.3 ± 3.0	0.483
Gestational age (weeks)	39.3 ± 0.9 ^ab^	39.0 ± 1.2 ^ab^	38.9 ± 1.0 ^a^	40.0 ± 2.2 ^b^	40.0 ± 2.1 ^b^	<0.05
Prepregnancy BMI (kg/m^2^)	20.5 ± 1.9	20.2 ± 2.1	20.9 ± 2.7	20.9 ± 3.1	20.2 ± 2.1	0.503
Predelivery BMI (kg/m^2^)	26.5 ± 2.5	25.4 ± 2.5	26.4 ± 2.7	26.9 ± 3.1	25.7 ± 2.6	0.115
Gestational weight (kg)	15.4 ± 5.1	13.3 ± 4.6	14.0 ± 4.3	15.1 ± 5.7	13.9 ± 4.5	0.343
Vaginal delivery	17 (56.7%)	16 (53.3%)	23 (47.9%)	20 (39.2%)	25 (54.3%)	0.489
Offspring gender						
Male	14 (46.7%)	18 (60.0%)	24 (50.0%)	31 (60.8%)	26 (56.5%)	0.631
Female	16 (53.3%)	12 (40.0%)	24 (50.0%)	20 (39.2%)	20 (43.5%)	
Offspring weight (g)	3372.7 ± 315.1	3331.0 ± 323.7	3416.8 ± 374.6	3531.1 ± 864.8	3379.1 ± 785.0	0.347

Data are expressed as means ± standard deviations for continuous variables and proportions for categorical variables. Here, *p* values (2-sided) were derived using ANOVA (continuous variables) and Chi-squared tests (categorical variables); Values that do not share a lowercase letter are significantly different. *p* < 0.05 indicates a significant difference during different lactating periods. BMI = body mass index.

**Table 2:** Concentration of breast milk folate over 1–400 days postpartum (ng/mL).

Folate	Days Postpartum (*n*)				
	1–5 Days (*n* = 30)	10–15 Days (*n* = 30)	40–45 Days (*n* = 48)	200–240 Days (*n* = 51)	300–400 Days (*n* = 46)
5-MTHF	8.52 (7.51,10.07) ^c^	20.24 (17.55,23.02) ^b^	38.06 (22.91,49.01) ^a^	40.65 (32.58,50.17) ^a^	40.49 (27.00,48.73) ^a^
UMFA	0.60 (0.51,0.71) ^b^	0.99 (0.81,1.27) ^a^	0.00 (0.00,0.66) ^c^	1.24 (0.94,2.99) ^a^	1.06 (0.82,8.25) ^a^
The minor reduced folate	3.48 (2.71,4.65) ^d^	6.83 (5.49,11.07) ^bc^	16.15 (11.39,24.71) ^a^	9.89 (5.80,14.05) ^b^	11.07 (7.81,15.71) ^b^
Reduced folate	12.09 (10.39,14.67) ^c^	27.52 (23.18,32.02) ^b^	54.65 (35.91,71.62) ^a^	51.29 (41.45,62.48) ^a^	52.71 (34.54,65.38) ^a^
Total folate	12.86 (10.91,15.58) ^c^	28.42 (26.09,32.97) ^b^	54.65 (37.42,71.94) ^a^	56.77 (44.83,73.92) ^a^	55.14 (35.78,70.58) ^a^

Data are expressed as median values with interquartile range deviations for continuous variables. Note: *p* values (2-sided) were derived using the Kruskal–Wallis test, and Bonferroni correction was used for multiple comparisons if there were differences between groups. Values that do not share a lowercase letter are significantly different; *p* < 0.05 indicates a significant difference during lactational periods. Total folate represents the sum of UMFA, THF, 5-methylTHF, 5,10-methenyl-THF and 5-formyl-THF. Reduced folates represent the sum of THF, 5-methylTHF, 5,10-methenyl-THF and 5-formyl-THF. The minor reduced folate represents the sum of THF, 5,10-methenyl-THF and 5-formyl-THF. THF, tetrahydrofolate; UMFA, unmetabolized folic acid.

## Data Availability

The data are available in a publicly accessible repository.

## Supplementary Materials

The following are available online at https://www.mdpi.com/article/10.3390/nu14142962/s1, Table S1: Comparing the daily consumption between summer and winter groups over the last month of common food, Table S2: Concentration of breast milk folate over 1–400 days postpartum of supplement nonusers, Table S3: Concentration of breast milk folate over 1–400 days postpartum of supplement users.
