# Supplementary material for: Profile of Folate in Breast Milk from Chinese Women over 1–400 Days Postpartum
Source: Nutrients. 2022 Jul 20;14(14):2962. doi: 10.3390/nu14142962 (PMC9319857; doi:10.3390/nu14142962)
Supplement: Supplementary file 1 [file nutrients-14-02962-s001.zip › nutrients-1756400-supplementary.pdf]

Supplemental (Tables 1–3)

Supplemental Table S1. Comparing the daily consumption between summer and winter groups over the last month of common food

Food item/category	Summer(*n*=54)	Winter(*n*=57)	*P* value
Cereal(g/d)	367.9	377.1	>0.05
Potato(g/d)	42.9	64.3	>0.05
Beans/dry peas(g/d)	35.4	50.0	>0.05
Vegetables(g/d)	315.7	271.4	>0.05
Fungus, Algae(g/d)	12.5	14.8	>0.05
Fruits(g/d)	121.4	115.0	>0.05
Livestock meat(g/d)	117.6	114.3	>0.05
Poultry meat(g/d)	28.6	14.3	>0.05
Milk and dairy products(g/d)	250.0	250.0	>0.05
Eggs(g/d)	50.0	50.0	>0.05
Fish and shrimp and a crab(g/d)	39.8	35.7	>0.05

Data represent the median daily consumption of dietary adjusted for portion size. Differences in consumption of dietary between summer and winter groups were assessed by Mann-Whitney Rank Sum test; $P < 0.05$ was considered significant.

Supplemental Table S2 Concentration of breast milk folate over 1-400 days postpartum of supplement nonusers

Folate	Days postpartum(n)				
	1-5d(*n*=19)	10-15d(*n*=22)	40-45d(*n*=43)	200-240d(*n*=34)	300-400d(*n*=28)
5-MTHF	8.61(7.70,10.49) ^{a}	19.34(16.85,23.02) ^{a}	38.39(24.52,49.02) ^{b}	43.42(33.09,52.70) ^{b}	41.50(28.66,49.38) ^{b}
UMFA	0.58(0.47,0.69) ^{a}	0.99(0.76,1.38) ^{b}	0.00(0.00,0.00) ^{a}	1.06(0.90,1.70) ^{b}	0.90(0.79,1.29) ^{b}
The minor reduced folate	3.79(2.93,4.88) ^{a}	7.11(5.27,11.07) ^{ab}	16.28(11.67,24.74) ^{d}	8.76(5.70,11.94) ^{bc}	11.48(9.56,15.54) ^{cd}
Reduced folate	12.09(10.81,14.84) ^{a}	27.52(22.11,32.02) ^{a}	55.08(37.48,72.66) ^{b}	52.42(41.69,67.91) ^{b}	55.63(39.86,65.96) ^{b}
Total folate	12.77(11.41,15.56) ^{a}	28.25(23.02,32.97) ^{a}	55.08(37.48,72.66) ^{b}	55.28(44.64,69.41) ^{b}	57.89(41.97,68.32) ^{b}

p values (2-sided) were derived using the Kruskal–Wallis test, and Bonferroni correction was used for multiple comparisons if there were differences between groups. Values that do not share a lowercase letter are significantly different; $p < 0.05$ indicates a significant difference during lactational periods.

Supplemental Table S3 Concentration of breast milk folate over 1-400 days postpartum of supplement users

Folate	Days postpartum(n)				
	1-5d(n=11)	10-15d(n=8)	40-45d(n=5)	200-240d(n=17)	300-400d(n=18)
5-MTHF	7.92(7.24,9.93) [a]	20.94(19.67,24.35) [ab]	23.64(20.40,66.02) [a]	36.54(29.94,44.89) [a]	32.99(22.30,46.88) [a]
UMFA	0.61(0.52,1.06) [a]	1.02(0.84,1.23) [ab]	9.20(0.75,23.82) [ab]	12.84(1.19,41.28) [b]	6.22(1.04,14.21) [b]
The minor reduced folate	3.31(2.50,4.24) [a]	6.80(5.80,11.76) [ab]	12.41(7.17,28.65) [b]	11.77(7.01,15.66) [b]	8.78(6.36,16.35) [b]
Reduced folate	11.89(10.09,13.58) [a]	27.45(26.44,40.40) [ab]	30.57(30.30,94.66) [b]	49.53(36.13,61.98) [b]	42.66(29.04,65.38) [b]
Total folate	12.95(10.60,15.63) [a]	28.53(27.33,46.24) [ab]	39.73(31.08,118.47) [b]	63.55(46.09,92.86) [b]	49.47(33.94,79.32) [b]

*p v*alues (2-sided) were derived using the Kruskal–Wallis test, and Bonferroni correction was used for multiple comparisons if there were differences between groups. Values that do not share a lowercase letter are significantly different; $p < 0.05$ indicates a significant difference during lactational periods.
